# The Effect of Severe Shot Peening on Fatigue Life of Laser Powder Bed Fusion Manufactured 316L Stainless Steel

**DOI:** 10.3390/ma15103517

**Published:** 2022-05-13

**Authors:** Timo Rautio, Matias Jaskari, Tejas Gundgire, Terho Iso-Junno, Minnamari Vippola, Antti Järvenpää

**Affiliations:** 1 Kerttu Saalasti Institute, University of Oulu, Pajatie 5, 85500 Nivala, Finland; matias.jaskari@oulu.fi (M.J.); terho.iso-junno@oulu.fi (T.I.-J.); antti.jarvenpaa@oulu.fi (A.J.); 2 Materials Science and Environmental Engineering, Tampere University, 33014 Tampere, Finland; tejas.gundgire@tuni.fi (T.G.); minnamari.vippola@tuni.fi (M.V.)

**Keywords:** severe shot peening, laser powder bed fusion, 316L, bending fatigue

## Abstract

Severe shot peening (SSP) was used on additive manufactured 316L by laser powder bed fusion. The effect of the post processing on the surface features of the material was analyzed through residual stress measurements, tensile testing, hardness-depth profiles, and fatigue testing by flexural bending. The results showed that SSP can be utilized to form residual stresses up to −400 MPa 200 μm below the surface. At the same time, a clear improvement on the surface hardness was achieved from 275 HV to near 650 HV. These together resulted in a clear improvement on material strength which was recorded at 10% improvement in ultimate tensile strength. Most significantly, the fatigue limit of the material was tripled from 200 MPa to over 600 MPa and the overall fatigue strength raised similarly from a low to high cycle regime.

## 1. Introduction

Additive manufacturing (AM) has successfully challenged traditional manufacturing methods in many industries, most notably on aerospace, biomedical and automotive areas [1,2,3]. This has been enabled by some of the key benefits that the technique provides: the ability to design complex parts without additional cost, reduction in weight and the low cost of manufacturing unique products. From a material perspective, austenitic stainless steel 316L is one of the most prominent materials currently utilized in the AM sector and the microstructure of the laser powder bed fusion (LPBF) manufactured 316L has been widely studied [4,5]. The results have showed that the mechanical properties of the LPBF manufactured material in terms of the strength exceed the properties of traditionally manufactured peers [6]. However, the typical trade-off between the strength and ductility must be noted. This work turns the focus on improving the material properties further with the aid of severe shot peening (SSP).

The fatigue strength of a given material is in general defined by the defects originating from the manufacturing process whether it is casting, forging or AM. The defect types and sizes vary between the processes and should be considered when high fatigue life is desired. Defect types vary also between the different AM techniques and for example the defect shape in LPBF manufactured parts is usually irregular, while, in binder jet printing (BJP), it is more spherical in nature [7]. Numerous studies have been published on the effect of defects to fatigue resistance of AM materials. For example, Solberg et al. [8] studied the effect of porosity to axial fatigue behavior of AM AISI 316L. They found out that the inner defects were responsible for poor fatigue resistance, crack initiation location depending on used stress amplitude, and a fatigue limit of 163 MPa at 2 million cycles was reached. Zhang et al. [9] argued that the effect of defects can be minimal, if the pore size and distribution were relatively low and the laser power were differentiated within 30% from optimal, causing the majority of cracks to initiate from slip bands. Their results showed a fatigue limit of 350 MPa with R = 0.1 and a run-out of 1 million cycles for the 316L. In bending fatigue, the effect of surface and near surface properties are promoted, as the maximum strains concentrate on the surface. A brief study about the fatigue behavior in bending fatigue of additively manufactured AISI 316L was published by Jaskari et al. [5], and they found out that the porosity in or near the surface dictated the fatigue behavior, regardless of the microstructure or achieved mechanical properties and presented a fatigue limit of 289 MPa at 2 million with R = −1 when higher energy density was used. In addition, the high cycle fatigue properties of the LPBF manufactured 316L in axial loading have been studied previously showing that an endurance limit of 568 MPa can be reached in the absence of critical defect using 10^6^ cycles and R = 0.1 [10].

In addition to the defects in the material, the surface properties of the material have a considerable effect on the fatigue life as the surface roughness can act similarly to a short crack on the surface [11]. Previous studies [12,13,14] have also shown that the defect distribution in LPBF parts is mainly focused on the near surface areas. Shot peening (SP) has been successfully utilized for different types of conventional austenitic stainless steels to create heavy surface deformation or even surface grain refinement up to nano-scale grain size for improved mechanical properties [15,16,17]. SSP can also induce martensite transformation on the surface [18]. In addition, increased surface hardness and rougher surface are usually accompanied with the process. However, the surface roughness resulting from the LPBF process in an as built condition is usually rough and can be improved with some suitable SP parameters and shot media. For example, in the authors’ previous work with LPBF manufactured AlSi10Mg, a reduction of R${}_{z}$ from 79 to 47 μm was reported after a glass bead SP [19]. It has been reported by Ahmed et al. [20] that the SP compromises the corrosion resistance of the material, but it can be controlled with the shot size to some degree. They also investigated the wettability of the material in terms of SP and found it could be improved.

Residual stress is an integral part of LPBF processed parts because of the lower process temperature, higher cooling rates as well as complex heating and cooling cycles [21,22]. The rapid heating induces compressive residual stresses because of constrained material expansion against the powder. Furthermore, higher temperature gradients during printing facilitate rapid cooling because of which material shrinkage takes place. This shrinkage is opposed by the surrounding powder which produces tensile residual stresses [23,24]. These complex residual stresses may lead to deformation and cracking which could result in detrimental mechanical performance. Hence, efforts are being made to control these residual stresses by optimizing different process parameters [21,25]. However, different post processing methods such as heat treatment, shot peening, and laser shock peening are also used to minimize the effect of these detrimental residual stresses [26]. Usually, for LPBF, processed stainless steel components are subjected to annealing to relieve these residual stresses [27]. In addition, the method like shot peening is used to induce beneficial compressive residual stresses on surface and subsurface. The presence of larger compressive stresses near the surface could facilitate improved fatigue life [28]. These residual stresses in LPBF samples could be tailored to larger compressive values near the surface with the help of SSP. Previously, Bagherifard et al. [29] have reported that subjecting AISI 316L stainless steel samples to SSP results in relatively larger compressive stresses and increased RS depth when compared with conventional shot peening. Liu et al. [18] also reported the similar behavior for 304 austenitic stainless steel when subjected to SSP.

The effect of SP on the bending fatigue performance of the LPBF manufactured 316L has not been previously investigated. The present study extends the application of the SSP process on the LPBF manufactured 316L and investigates the effect on the mechanical properties. In addition, the influence on the hardness, residual stresses and fatigue life in flexural bending will be demonstrated.

## 2. Materials and Methods

### 2.1. Material and LPBF Manufacturing

An SLM 280 HL machine (SLM Solutions Group AG, Lübeck, Germany) based on the LPBF technique was used to manufacture the samples required for this work. Spherically shaped 316L powder was supplied by Carpenter Additive of Carpenter Technology Corporation (UK) and had a particle size range of 15–45 μm and the chemical composition detailed in Table 1. Relevant printing parameters received from the machine supplier were as follows: laser power (*P*) of 200 W, speed (*v*) of 800 mm/s, hatch spacing (*h*) of 120 μm and layer thickness (*t*) of 30 μm. These values result in an energy density of 69.4 J/mm^3^ when applied to Equation (2):

(1) $$E=\frac{P}{v\cdot h\cdot t}.$$

**Figure 1:**
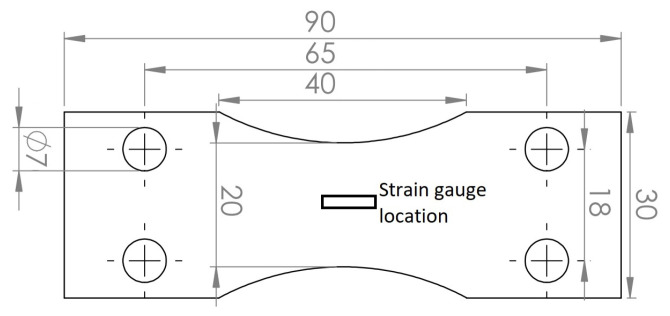
Specimen geometry in mm used for the bending fatigue tests.

To reduce the printing costs, horizontal orientation was chosen for the tensiles, but the fatigue specimens were printed in a vertical direction. The tensile specimens were printed in the size of 25 × 120 × 2 mm and the fatigue specimens were 30 × 90 × 2 mm. The DIN 50100 standard was used in preparing the specimens for fatigue and the dimensions with the strain gauge location are presented in Figure 1. Prior to the printing, the platform (280 mm × 280 mm) was heated to 150 °C and held constant during the printing. The printing process was carried out in an argon atmosphere at a pressure of 12 mbar and a gas flow of 7.5 m/s.

After the printing, residual stress removal was performed for the printed specimens by a stress relief heat treatment (HT) in a muffle furnace (Sarlin 1000HS-436, Sarlin Oy Ab, Vantaa, Finland). Specimens were held in constant 600 °C in argon atmosphere for two hours, followed by cooling to room temperature in the furnace overnight. Tensile and fatigue specimens were then machined to the final shape after the HT and SSP procedures. Used test length and width were 25 mm and 5 mm, respectively, for the tensile specimens, and the fatigue specimens were machined to their required hourglass shape.

### 2.2. Shot Peening

The shot peening process was carried out in a customized closed cabinet with the aid of a six-axis industrial robot. A pressurized shot blasting pot was operated from the outside of the cabinet with the nozzle fixed to the cabinet front face. This nozzle was specifically designed to achieve the desired coverage shape. Considering the wear during the SP, several identical pieces were manufactured with the LPBF printer.

A special holder for the specimen under treatment was built to manage the repeated shot peening processing on its both sides. For the precise control and repeatability of the process, the shot peening time and specimen movement was controlled by the robot operating on the holder. After each pass, the specimen was rotated 180° to increase the intensity evenly on both sides and the process was repeated for a maximum of 22 times per each side.

Spherical martensitic chromium media (STELUX C40) were selected for the SP experiments in the size range of 0.30–0.85 mm and hardness of 36 HRC when new. Almen intensity measurements were adopted from the beginning to determine the effectiveness of the process and to monitor the process variations (wear on nozzle and SP media, etc.). This process was conducted according to [30]. The time scale of the measurement was calculated as number of passes past the nozzle. Preliminary studies were conducted to find the most effective distance from the nozzle, the nozzle size as well as the optimal pressure. Based on these, tensile and bending fatigue samples were blasted with an operating pressure of 7.4 bar and 70 mm from the SP nozzle.

**Figure 2:**
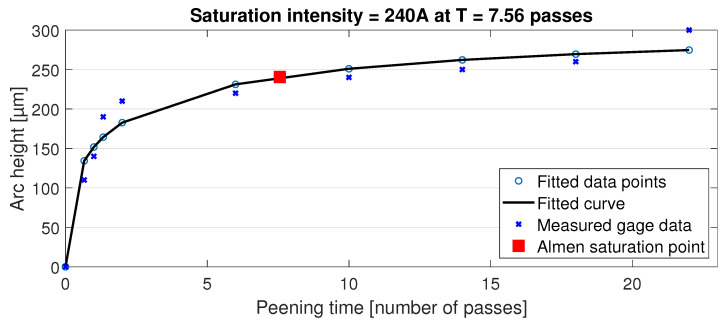
Almen intensity measurement.

Almen intensity was determined with type A strips using a range of passes from 1 to 22 at the speed of 50 mm/s and the resulting data points with a fitted curve are presented in Figure 2. By definition, the Almen intensity is the point where reducing the SP time by 50% has resulted in a 10% drop in the arc height of the Almen strip. This point in the chart is shown by the red square representing an Almen intensity of 240 A. The actual SP of the specimens was carried out using 22 passes to gain highest surface modification possible for this shot media and test arrangement.

### 2.3. Residual Stress and Roughness Measurements

**Figure 3:**
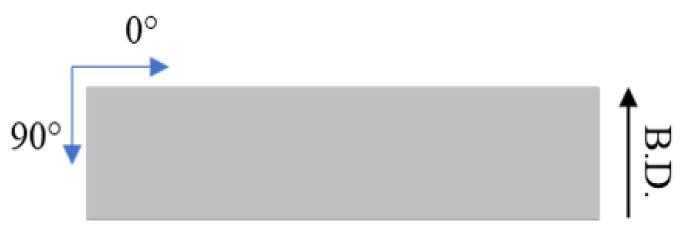
Residual stress measurement directions. The building direction is indicated by a black arrow.

The residual stress measurements were performed on XStress 3000 equipment (Stresstech Oy, Jyväskylä, Finland) with the modified Chi method. The modified chi method uses the X-ray diffraction mechanism with two X-ray detectors placed symmetrically on the either side of the incident beam. The method calculates inter-planar lattice distances which could be either compressed or stretched out depending on the type of residual state at the corresponding location [31]. The measurements were performed using a Manganese (Mn) tube with a diffraction angle of 152.3°. A collimator with a 3 mm diameter was used for the measurements. Stresses were measured both in longitudinal (0) and tangential (90) direction as shown in Figure 3, i.e., along the length and width of the sample, respectively. For the residual stress depth profile, electrolytic polishing was used to selectively remove a thin layer of material between each measurement. The depth after each step was measured using a dial gauge. Previously, the electrolytic polishing has been proven effective as well as reliable for the measurement of residual stresses along depth for the shot peened steels [32]. Valiorgue et al. [33] also reported this an effective method to measure the residual stress depth profiles without affecting the stress distribution. The surface roughness was measured with an optical profilometer (Alicona InfiniteFocus G5, Graz, Austria) by scanning an area of 4 mm × 4 mm. The average roughness (Ra) values were derived from the scanned area.

### 2.4. Characterization of Static and Dynamic Properties

Tensile testing was conducted according to the standard SFS-EN ISO 68921:2016 with a constant loading rate of 0.5 mm/min using an Instron universal material testing machine (Instron 8802, Norwood, MA, USA). An extensometer was not available for the tests, but the stress was measured in relation to the total elongation that can still be used to compare the structures reliably. The results were validated by four repetitions for all three of the structures, as built, HT and SSP. The as built and HT samples had the surface in an as printed condition without surface modifications. For the indentation hardness measurement of the shot peened structure, a cross-sectional sample was cut and polished. An indentation hardness depth profile was measured by 40 μm steps up to 0.52 mm depth using a 0.1 kg load and the process was repeated three times.

Fatigue properties of the studied structures were determined with a reversed flexural bending fatigue machine by Carl Schenck with stress ratio of R = −1 and frequency of 10 Hz. For this purpose, 2 mm thick specimens were manufactured in the standard hourglass shape with outer dimensions of 30 × 90 mm. Stress amplitudes range for the SP samples was 475–1100 MPa and 200–700 MPa for the as built and HT structures. Calibration with strain gauges was conducted for each structure separately before testing. Strain gauge is acting as a variable resistor on a Wheatstone bridge and the changes in length can be recorded as a corresponding voltage change that is measured and converted to strain. Applying Young’s modulus, the stress can be determined using Hooke’s law:

(2) σ=Eϵ.

where σ is stress, *E* is Young’s modulus and ϵ the strain. In bending, the maximum stress is at the surface and the results correspond to this. Temperature was kept at room temperature during the experiments with the aid of air cooling. The as built and HT samples did not have any surface modifications before the testing. The fatigue testing was carried out following the general principles for fatigue testing of metals defined in standard SFS 3099.

After fatigue testing, fracture surfaces were recorded by macroscopic imaging. More detailed analysis was conducted for selected samples using field emission scanning microscope (FESEM, Zeiss Sigma, Zeiss, Jena, Germany). Used acceleration voltage was 5 kV, the working distance was 10 mm, and secondary image (SE) detector was used for imaging.

## 3. Results

### 3.1. Tensile Strength

**Figure 4:**
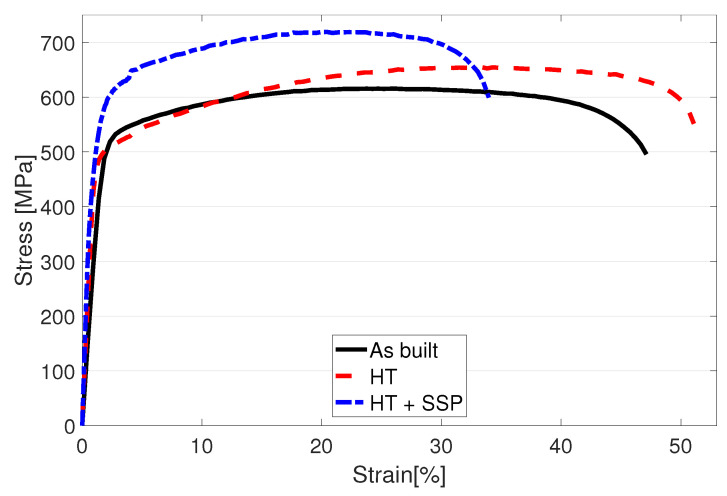
Tensile test results for the as built, heat treated and severe shot peened specimens of LPBF manufactured 316L steel.

Measured engineering stress curves for as built, HT and SSP after HT conditions are presented in Figure 4. and the obtained values of yield strength (YS) for 0.2%, 0.5% and 1% proof strengths, ultimate tensile strength (UTS) and elongation at break are collected in Table 2. In the as built condition, the base material had YS and UTS of 488 MPa and 615 MPa, respectively. After the HT, the material showed 4% lower YS but 6.3% higher UTS with a higher strain hardening rate. In addition, 8.4% improvement in ductility was also gained with the HT.

The structure after the HT and the subsequent SSP led to an engineering stress curve at a notably higher level compared to the as built and HT structures as shown by the dashed blue curve in Figure 4. While the YS for 0.2% proof strength had only a subtle 2.4% increase, the UTS was measured 10% higher compared to the HT condition. Visual examination of Figure 4 suggests that the YS should be the highest with the SSP after HT condition. Numerical analysis confirms this when 0.5% or 1% proof strength is used to define the YS. For example, with 1% proof strength, the YS of HT + SSP condition is 10.5% higher compared to as built material. Improved strength typically leads to some losses in the ductility, and this was also recorded here as a reduction from 42.6% to 33.9%.

### 3.2. Hardness

**Figure 5:**
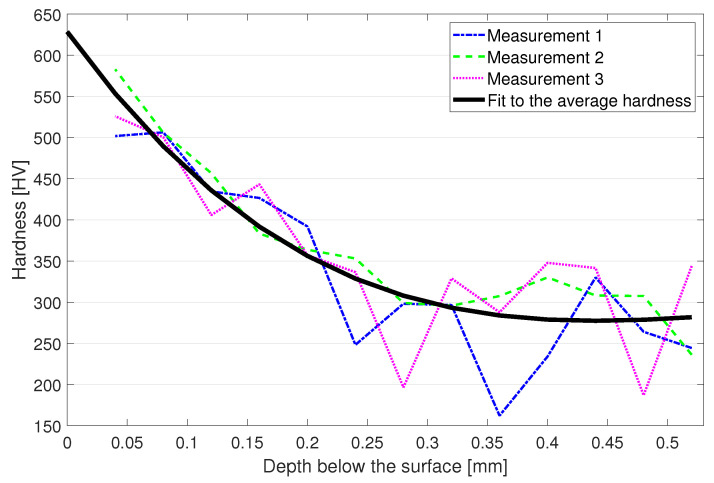
Hardness-depth profile of the heat treated and severe shot peened structure.

Hardness-depth profile of the SSP 316L after HT is displayed in Figure 5. All three repetitions of the profile measurement showed a similar shape where the peak hardness was evidently at the measurement points nearest to the surface around 550 HV. From there, the hardness started to decrease with the highest rate and was 350 HV at 0.2 mm below the surface and continued to soften at a gradually lowering rate finally settling to the printed base material hardness of 275 HV at the depth of 0.4 mm. A 3rd degree polynomial curve was fitted to the average measurement data and the extrapolation to the surface direction suggests that the maximum hardness was below 650 HV.

### 3.3. Residual Stresses

**Figure 6:**
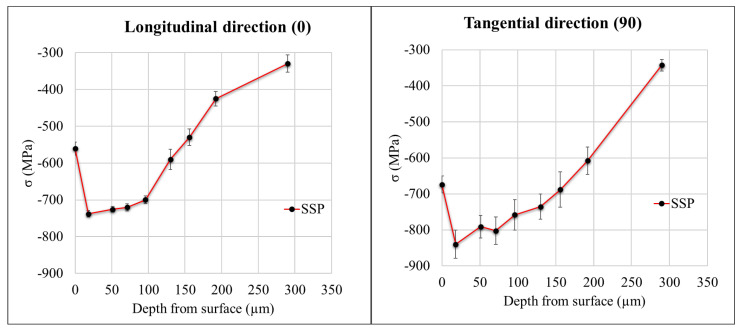
Residual stress depth profile for the severe shot peened sample (SSP).

The residual stress depth profile was measured for the SSP sample and is indicated in Figure 6. The surface residual stress was found to be around −550 MPa along the longitudinal (0°) direction and −670 MPa along the tangential (90°) direction. The compressive residual stresses kept increasing along the depth and remained more than −700 MPa up to 100 μm depth and −400 MPa up to 200 μm depth along both the directions.

### 3.4. Bending Fatigue

**Figure 7:**
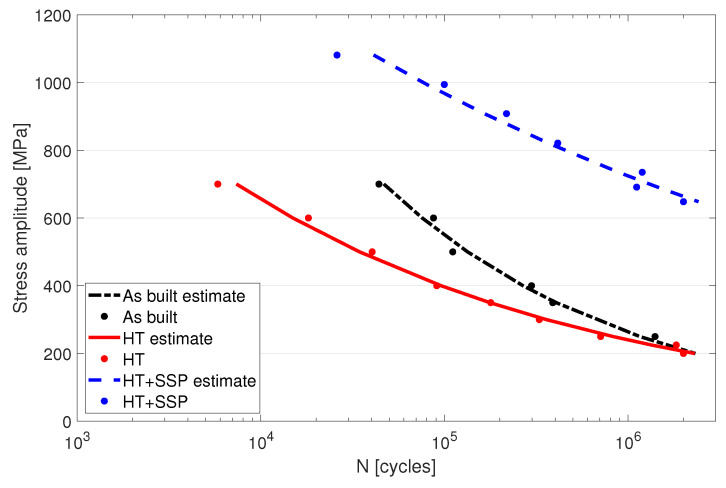
Stress amplitude and the corresponding fatigue lives for as built, heat treated, and severe shot peened after heat treatment conditions.

The fatigue properties of the three different conditions of the LPBF manufactured 316L were analyzed up to the high cycle fatigue range (run out at 2 million cycles) with a reversed flexural bending fatigue machine. The specimen lifetime related to the stress amplitude are displayed by the conventional S-N curves in Figure 7. The results showed that the as built and HT conditions exhibited identical lifetimes in high cycle regime withstanding 2 × 10^6^ cycles at 200 MPa stress. In the low cycle regime (<10^5^), the as built material outperformed the HT material and withstood nearly 10 times the cycles at 700 MPa stress level as suggested by the lower yield strength of the HT condition.

Utilizing SSP after the HT resulted in a remarkable improvement in the fatigue life of the parts as can be seen on the blue dashed line in Figure 7. Higher durability was gained in both low cycle and high cycle regimes making the SSP condition superior compared to the other conditions. Fatigue limit for the SSP sample was measured at a little over 600 MPa, which was over three times higher compared to the 200 MPa of the HT condition. Stress levels up to 1100 MPa could be conducted with the equipment, and at that point the fatigue life was at 26,000 cycles.

**Figure 8:**
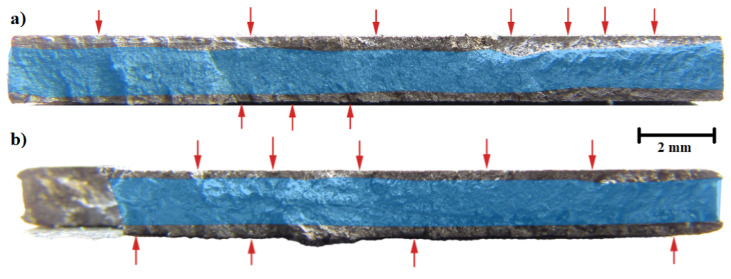
Fracture surfaces of the heat treated (**a**) and severe shot peened after heat treatment (**b**) samples. Red arrows indicate fracture initiation sites, and the blue areas are estimates of the final fracture area.

**Figure 9:**
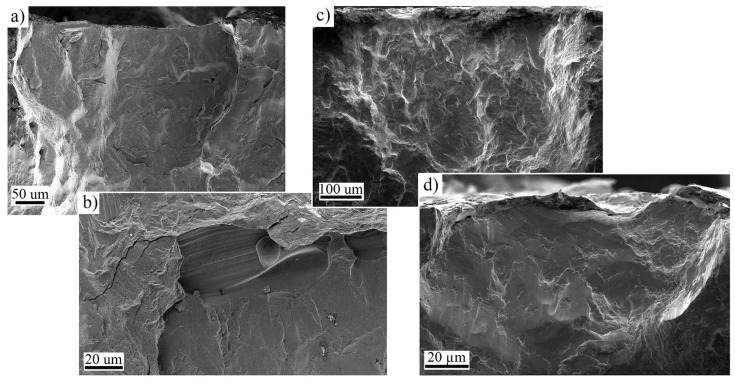
SE-images showing the typical crack initiation sites in fractured samples. For As built and HT sample, fracture initiated either from the (**a**) surface or from (**b**) lack of fusion defects. SSP samples fractured mainly from surface irregularities (**c**,**d**).

As an example of the high cycle regime fracture behavior, the macroscopic fracture surface images of samples after HT and HT + SSP with similar fatigue lifetimes are presented in Figure 8. Used stress amplitudes were 200 and 690 MPa for HT and HT + SSP, respectively, and the lifetimes were around 1 × 10^6^ cycles. It can be noted how the fracture initiated from several locations on the surface in both cases (red arrows in Figure 8), and initiation sites could be found on both surfaces. Although the behavior was similar, the crack propagation depth is somewhat smaller for HT + SSP sample (blue areas in Figure 8). Closer inspection of fracture surfaces presented in Figure 9 revealed the typical initiation sites to be either the lack of fusion type defects, or surface irregularities for the as built and HT samples. For SSP samples, only surface initiation sites were observed, and the failures initiated from surface irregularities, such as notches and grooves.

## 4. Discussion

While the AM technique has been already adopted in many industries, the cost of the manufacturing is still limiting the growth of the technique on many areas. Currently, AM is still not a viable replacement for all manufacturing technologies but should be capitalized in the areas where its superior properties can be utilized to produce higher quality products compared to other techniques. The strength of the LPBF manufactured 316L already surpasses the sheet metal counterparts [6] without any additional post processing. However, the fatigue properties are susceptible to lower surface quality and unfavorable defect distribution of the LPBF printed material. The results presented in this paper show that the SSP can be successfully used to improve the fatigue life of LPBF manufactured parts.

One of the contributing qualities gained with the SSP is the increased surface hardness that is generally accompanied with an increase in the fatigue life. The surface roughness also plays an important role on the fatigue life [34], and SP can be used to increase the surface quality of LPBF printed parts [19]. It is worthwhile to mention that SSP has reduced the average roughness (Ra) value to 3.93 ± 0.006 μm from 8.81 ± 0.059 μm of the HT sample. The base material hardness was measured around 225 HV with macroindentation tests in the authors’ previous study [35]. Microhardness measurements usually give a little higher result, and this was also the case here as the microhardness level was measured at 275 HV. The Almen intensity of 240 A utilized in this work generated a surface hardness of over 600 HV which is considerably higher than the base material and more than 50% higher compared to the results in [20] with a higher Almen intensity of 280 A. These results suggest that a heavier and harder shot with a comparable intensity can be used to reach improved hardness levels at the surface as well as a higher impact on the hardness-depth profile underneath it. SMAT (Surface Mechanical Attrition Treatment) is generally utilized to gain very high compressive stresses at the surface similarly to SSP, but the results show that a low cost SSP can be used to gain improved results in terms of surface hardness (334 HV at the surface [36]). In addition to hardness, the strength properties were elevated after SSP, and it is consistent with findings in literature. SSP causes massive deformation near the surface, and the effect is proportional to the shot peening media size and velocity [21].

**Figure 10:**
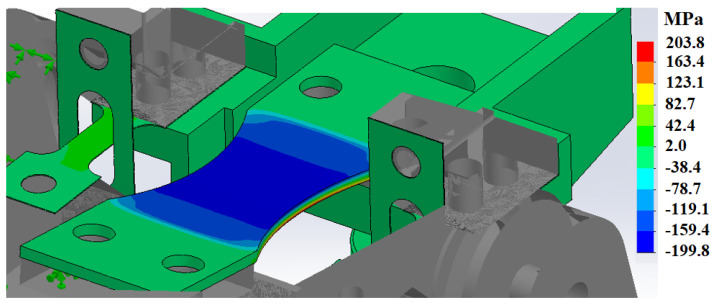
Stress gradient on the specimen under stress of 200 MPa.

**Figure 11:**
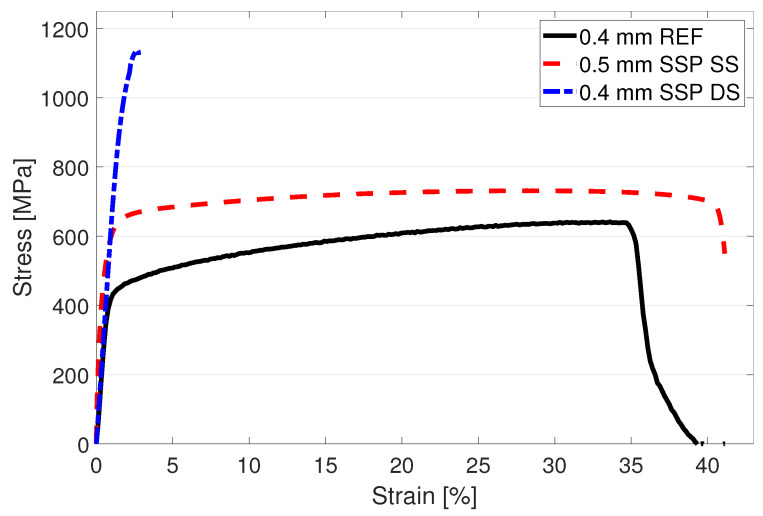
Tensile strength of a thin 316L sheet metal and after subjecting to SSP treatment for single side (SS) and both sides (DD).

The fatigue results presented in Section 3.4 showed low cycle fatigue strength reaching 1100 MPa, which is clearly higher than the tensile strength of the same material. In flexural bending, the surface experiences the highest amount of stress, which gradually lowers to zero at the neutral axis in the mid-thickness of the specimen. This is clarified in Figure 10 where the upward bending has created a compressive stress of 200 MPa on the upper surface and tension of similar magnitude on the lower surface while the neutral axis at the mid-thickness remains free from stress. In axial fatigue testing, the whole cross-section of the specimen is subjected to a uniform stress, pointing out a clear difference between the testing methods. The aforementioned leads to the fact that increased strength at the surface facilitates the bending fatigue testing at higher levels than the base material strength proposes and also gives higher fatigue strengths compared to axial fatigue testing as a lower volume of the specimen experiences the maximum stress applied. To further validate the results, wire-EDM was used to cut thin slices of 0.4 mm and 0.5 mm thick from a 316L sheet metal which was SSP processed from one side for the 0.5 mm sample and both sides for the 0.4 mm sample. Subsequent tensile testing (Figure 11) revealed the markedly higher tensile strength of the SSP processed material. The hardness profile presented in Figure 5 indicates that the hardness of a 0.5 mm thick plate is not modified in full thickness, but the tensile strength still shows clear improvement. Double sided SSP treatment modifies the material almost thoroughly raising both surfaces up to 600 HV and the middle to approximately 350 HV. This increases the ultimate tensile strength already to 1150 MPa, and a uniform 600 HV hardness in the specimen would increase it even further.

The effect of SSP can be clearly seen on residual stress behavior of the sample (refer Figure 6). The sample shows the higher compressive residual stresses of more than −700 MPa at 100 μm depth beneath the surface, which is consistent with previous study by Lui et al. [18]. Furthermore, the compressive stresses of more than −400 MPa were observed even at 200 μm depth beneath the surface, which is higher than the previous findings [29,37]. These results strengthen understanding that SSP induces larger compressive stresses on the surface and beneath. Furthermore, it also induces compressive residual stresses deeper into the sample when compared with the conventional shot peening. With defects in LPBF parts mainly being concentrated in near surface vicinity [12,13,14], SSP shall facilitate improved fatigue performance of the material, which is discussed below.

The fatigue strength of the specimens without surface modification is most likely limited by the porosity and surface roughness, as the results were nearly identical at roughly 200 MPa. On the low cycle regime, the fatigue strength is mainly determined by the yield strength of the material resulting in better performance of the as built material compared to the HT counterpart. The surface modification with the increased hardness and compressive stresses by SSP had a clear impact on the strength properties and also resulted in a higher strain hardening rate. Similarly, in the work by Li et al., they found improved hardness profile and increased YS after a rotationally accelerated shot peening on a 316L sheet metal [38]. However, in this work, the most prominent effect was seen on the increased fatigue life of the specimens. It is well known that the porosity greatly reduces the fatigue life on high cycle regime also with the AM parts [7]. Previous studies have also shown that the fatigue crack in AM 316L initiates favorably from the lack of fusion faults near the surface [34], and it seems to also apply in this study for the as built and HT samples. The results of the present study suggest that the SSP could be used to eliminate the porosity under the surface. This together with the increased hardness and compressive stress level resulted in a considerably higher fatigue strength. It should be noted that the bending fatigue testing pronounces the effect of surface defects, and the presence of them would lower the results. In addition, higher yield strength of the SSP specimen also prolongs the crack nucleation period that shows on the low cycle regime, although the crack growth area can be smaller due to a hard surface layer. Even though the crack initiation and propagation are inhibited, due to high stress amplitudes, the crack propagation increases exponentially after the crack has propagated through the hardest layers.

## 5. Conclusions

The effect of using severe shot peening (SSP) on additively manufactured 316L by laser powder bed fusion was analyzed in this work with the following conclusions:

- SSP induced beneficial larger compressive residual stresses of more than 700 MPa at 100 μm depth along with reduced surface roughness;
- Increased mechanical strength was achieved with the SSP leading to an ultimate tensile strength increase of 10%;
- Hardness-depth profile measurements revealed a maximum hardness of nearly 650 HV at the surface after the SSP compared to 275 HV of as built material at the depth of 0.4 mm;
- Bending fatigue performance of the material was notably increased with the fatigue limit recorded at 600 MPa after the SSP compared to the 200 of as built material.

## Figures and Tables

**Table 1:** Chemical composition of the 316L powder in wt% utilized in this work.

Fe	Ni	Cr	Mn	Mo	C	Si	Cu	N	O	S	P
Balance	12.5	17.6	0.66	2.38	0.02	0.65	0.02	0.09	0.03	0.006	0.007

**Table 2:** Mechanical properties of the printed 316L structures: yield strength (YS) for proof stress of 0.2%, 0.5% and 1%, ultimate tensile strength (UTS) and elongation at break.

	YS 0.2% [MPa]	YS 0.5% [MPa]	YS 1% [MPa]	UTS [MPa]	Elongation at Break [%]
As built	488 ± 2.2	520 ± 2.3	533 ± 2.3	615 ± 1.4	39.3 ± 0.2
HT	468 ± 2.1	496 ± 2.2	503 ± 2.2	654 ± 2.0	42.6 ± 0.3
HT + SSP	479 ± 1.2	535 ± 1.3	589 ± 1.5	719 ± 2.3	33.9 ± 0.4

## Data Availability

Data sharing is not applicable to this article.

## Abbreviations

The following abbreviations are used in this manuscript:

AM	Additive Manufacturing
BF	Bending Fatigue
LPBF	Laser Powder Bed Fusion
SP	Shot Peening
SSP	Severe Shot Peening
